# Minutes of the Twenty-Third Annual Session of the Southern Dental Association

**Published:** 1892-01

**Authors:** 


### AMERICAN JOURNAL

—OF—

### DENTAL SCIENCE.

VOL. XXV. BALTIMORE, JANUARY, 1892. NO. 9.

ARTICLE I.

### MINUTES OF THE TWENTY-THIRD ANNUAL SESSION OF THE SOUTHERN DENTAL ASSOCIATION.

HELD IN THE NORTH CAROLINA TEACHERS' ASSEMBLY HALL AT MOREHEAD, N. C., AUG. 11, AT 10.30 A. M.

### MORNING SESSION.

The meeting was called to order at 10:30 a. m., with Dr. G. F. S. Wright, of Georgetown, S. C., in the chair.

The exercises were opened with prayer by the Rev. Dr. Stebbins, of Raleigh, N. C.

After which, Dr. H. C. Herring introduced Dr. J. F. Griffiths, of North Carolina, who delivered the welcoming address.

After the calling of the roll by the secretary, Dr. M. C. Marshall of Little Rock, Ark., Dr. J. F. Calvert, of South Carolina, presented the president with an ivory, gold and ebony gavel, which was accepted.

The president next read his opening address as follows:

*Officers and Members of the Southern Dental Association, Ladies and Gentlemen:*—The pleasure of meeting you again, the pleasure of hearing the eloquent words of welcome to North Carolina, to Morehead City, and the musically voiced acceptance, have been heard. Also, that greatest of all that is grand and glorious, the honor of worshipping Deity, has been ours.

We are grateful; but *all* are *not* here. Some detained at home by force of circumstances, others are visiting different scenes. These, we hope to meet again. Others have gone to the God they worshipped here.

RECORDS—PUBLISHED AND UNPUBLISHED.

At our last meeting, my predecessor in his able address alluded to the disordered condition of our history.

Having in my possession the published proceedings for the years 1870, '72 and '73, from *these* and with the aid of our recording secretary, I have collected the following, hoping it may interest you, and believing that "to be reminded of what is already known, is as important as to be taught something new."

ORGANIZATION.

The Southern Dental Association organized at Atlanta, Ga., in July, 1869, and Dr. B. F. Arrington was elected president. We have seen no published records of this meeting.

First meeting in New Orleans was in 1870, April 13-16 inclusive. Vice-President Dr. J. G. McAnaly, of Selma, Ala., presided. At this meeting fourteen members responded to roll call. Twenty-eight new members were elected.

Dr. J. S. Knapp, of Louisiana, was made president. Adjourned to meet in Charleston, S. C., where it was called to order by the president, April 12, 1871. He presided during this meeting. We know of no published records of that session, the recording secretary, Dr. J. G. Angel, of New Orleans, having died.

Dr. F. S. Clark, of Savannah, Ga., was elected to preside at the meeting held in Richmond, Va., in July, 1872.

Next, Dr. H. M. Grant, of Virginia, was elected. He presided at Baltimore in July, 1873.

The next president elected was Dr. Robert Arthur, and St. Louis, Mo., chosen for the place of meeting in 1874.

I do not think Dr. Arthur presided, but Dr. James Johnston of Staunton, Va., the second vice-president.

Our secretary informs me that there are no records for 1875-76.

In April, 1877, the Southern Dental Association met in Montgomery, Ala., Dr. S. J. Cobb, of Nashville, Tenn., president. A joint meeting was held this year at Oakland, Md. No new officers were elected.

August, 1878, met at Niagara Falls, where Dr. F. J. S, Gorgas was elected president.

In July, 1879, met in Augusta, Ga. Changed the name to National Dental Association. Elected Dr. J. B. Patrick, of Charleston, S C., president.

Met July, 1880, in New York City, where Dr. V. C. Turner, of North Carolina, was elected president and Ashville, N. C., selected for the place of meeting in July, 1881. Since that time it has been known as the *Southern* Dental Association.

Next met:

August, 1882, at Baltimore, Md., Dr. E. S. Chisolm, Alabama, presiding.

August, 1883, at Atlanta, Ga., Dr. L. D. Carpenter, Ga., presiding.

July, 1884, at Lexington, Ky., Dr. H. J. McKellups, Mo., presiding.

May, 1885, at New Orleans, La., Dr. A. O. Rawles, Ky., presiding.

August, 1886, at Nashville, Tenn., Dr. W. C. Wardlaw, Ga., presiding.

August, 1887, Old Point, Va., Dr. W. W. Thackston Va., presiding.

August, 1888, Louisville, Ky., Dr. B. H. Catchings, Ga., presiding.

August, 1889, at Galveston, Texas, Dr. J. G. Crawford, Tenn., presiding.

July, 1890, at Atlanta, Ga., Dr. J. C. Story, Texas, presiding.

This, August, 1891, for the second time we meet in North Carolina.

RECAPITULATION.

Met once in Alabama.
Met four times in Georgia.
Met twice in Kentucky.
Met twice in Louisiana.
Met three times in Maryland.
Met once in Missouri.
Met twice in New York.
Met twice in North Carolina.
Met once in South Carolina.
Met two or three times in Tennessee.
Met twice in Virginia.

The two meetings held in 1877 will account for *this* being the twenty-third meeting in twenty-two years.

JOURNAL.

Referring to the published proceedings of 1870, I read the names of a committee on the publication of a Dental Journal.

This committee consisted of fourteen names, with Dr. A. F. McLain, of Louisiana, Chairman.

I do not know if this committee did much work or was discouraged.

In 1873, the published transactions record as follows: "For the last three yeare this Association has entertained the idea of having a journal published under its patronage and by its fostering care.

"To consummate this much desired object, Dr. Gorgas offers you the AMERICAN JOURNAL OF DENTAL SCIENCE. We hope this meeting will not adjourn until some definite action has been taken in this matter, or, at least, that it be recognized as the organ of this Association. And that the proceedings of this meeting be published in its columns and a copy containing said proceedings be forwarded to each member entitled to the same by the constitution.

"Thus relieving the secretary and publication committee of a great portion of their labor, and saving to this Association the expense of publishing its proceedings in the usual manner.

### SOUTHERN DENTAL JOURNAL.

Now, the *Southern Dental Journal* claiming to be the organ of the Southern Dental Association, I would suggest that the annual proceedings of this body be published exclusively in a single number, and that one copy of that number be mailed to each member in good standing. Said number to contain the committees for the next meeting; also the code of ethics, constitution, by-laws and rules of order.

Stereotyped plates of these last named may be obtaind at a moderate cost and can be used annually.

### DENTAL COLLEGES.

Of forty-five reputable dental colleges, eight have ceased to be, as have also a number of minor claimants of the right to confer degrees. Thirty-seven claiming to be reputable, are now in operation.

Of these surviving institutions, twelve were at work in 1875.

### INFLUENCE OF EXAMINING BOARDS.

About this time the labors of chartered dental associations began to exercise a powerful influence upon dental education.

Associations having been formed *before* dental colleges, *these* are to some extent the product of dental associations.

Between these two, there cannot be a healthy rivalry; nor can either monopolize the mission of the other.

The Association has not only produced the college, but sustained it; first, by recommending the college to the student, next by securing the enactment of laws creating examining boards, which compelled students to attend the college.

Thus increasing the demand for something better or a *supplement to* private instruction. As the result, schools have mutiplied. Not *always* sustaining the best standard —consequently the associations, through the examining boards, have determined that the standard be maintained. Not fearing that it will be required of them to hinder the further elevation in consequence of Chinese methods or endowed colleges.

The last commencement day ended the old order of things, with an unusual number of graduates.

Next year, if the three year plan is adhered to in good faith by the colleges, and sustained by the board of examiners, comparatively few will be licensed.

The following year, ('93) promises to produce the *real* doctors of oral surgery.

### THE HOME OR CHATAUQUA.

At the first meeting in this State, did not our Association appear homeless indeed?

Having been inspired to attempt the then impossible, it turned to you for shelter, was renamed Southern, and learned that we, with those of our way of thinking, were at home anywhere, more especially from Maryland to Texas.

Last year we were startled and thrilled by the idea of a permanent home. Again you find us in North Carolina, where all that can be desired in a health promoting climate and beauty of scenery richly abounds.

I favor this idea. If it is not carried so far as to prevent our visiting other States or places, when the good of dentistry or humanity demands it.

I will say no more of this. To the chairman of the committee to select a home, must be awarded the footprints which lead to our dental Chatauqua.

DENTAL LEGISLATION.

The constitutionality of dental legislation, upon which depends the prosperity of our colleges, and by which the examining boards are empowered—has been questioned; and some cases, I think, have been reported, in which these laws have not been sustained by the courts.

With your permission, I will read the address of Hon. J. L. Tribble on the subject, delivered by him before the South Corolina State Dental Association, at their last meeting, July 15, 1891.

*Mr. President and Gentlemen of the South Carolina State Dental Association:*—The subject upon which I have been requested to address you at this meeting of the State Dental Association demands no flights of fancy nor figures of speech; but, as it seems to me, needs only a plain practical statement of the law applicable to the subject of dentistry and the history and reason for such laws. After looking over the field, I seriously doubted my ability to present anything on the subject that would be of special interest to this Association, but I cheerfully submit this to you for what it is worth, hoping that you may find here and there a grain of wheat, while blowing away the chaff.

In the first place, those of you who are familiar with our statutes regulating the practice of dentistry in this State, know that, by Section 944, General Statutes, this Association is a body politic and corporate, with power to make by-laws not inconsistent with the constitution and laws of the State. Section 937 also requires that this Association shall select a Board of Dental Examiners for the terms of one, two, three, four and five years respectively, or until their successors shall have been elected; and by Section 941, one member of this board has the authority to grant license to practice dentistry until the next regular meeting of the board. The statute further provides, that each dentist shall keep a record of all cases treated in his pratice in accordance with the form prescribed by the board, and to furnish a copy to the patient if so desired. Section 2594 provides as follows: "Any person who shall in violation of Section 936, practice dentistry in this State for fee or reward, shall be liable to indictment, and, on conviction, shall be fined not less than fifty or more than three hundred dollars: provided, that nothing in this chapter shall be so construed as to prevent any person from extracting teeth."

On trial of such indictment, it shall be incumbent on the defendant to show that he has authority, under the law, to practice dentistry, to exempt himself from such penalty, but those practicing dentistry regularly prior to February 25th, 1875, the time of the approval of the Act, are expressly exempted from the operation of the statute, by Section 2595, General Statutes. Then the question arises, who are legally authorized to practice dentistry in this State? I answer, first, those who were in the regular practice prior to the 25th of February, 1875. Second, those who comply with Section 936, as amended at the regular session of 1887, Acts, page 788. Notice the language: "No person shall hereafter enter upon the practice of dentistry in this State unless he shall have obtained a license from the Board of Dental Examiners in the State of South Carolina." This language is very broad and has no words of doubtful construction, and we must assume that the legislature means exactly what it says, nothing more, nothing less; and any man who practices dentistry without the license is a violator of the law, and his qualifications and character have nothing to do in the matter, for Sections 943 and 2594 say he shall be liable to indictment, and on conviction, punished in a sum of not less than fifty or more than three hundred dollars, and if indicted, it is incumbent on him to show that it is not in violation of the law.

Section 938 provides: "The Board of Examiners shall meet annually at the time and place of the meeting of the South Carolina State Dental Association, giving thirty days notice in the public newspapers published in not less than three different places in the State; * * * shall prescribe a course of reading for those who study dentistry under private instruction; shall grant licenses to all applicants who undergo a satisfactory examination; shall keep a book in which shall be registered all persons licensed to practice dentistry in the State of South Carolina."

I have thus briefly outlined and quoted the statutes in this State applicable to the regulation of the practice of dentistry.

Law in its most comprehensive sense is defined by the great commentator as a rule of action; and in this sense the statutes as promulgated by the legislature is a rule prescribed for your action, as well as for every one who desires to practice dentistry in South Carolina.

All laws are supposed to be founded upon reason or necessity. The question then arises, what is the reason and where is the necessity for such a law in South Carolina? Why not let every man who can handle a gouge or forceps practice dentistry as he pleases, or as he can find dupes or fools to practice on? Well, in certain quarters, parties insist we need protection; and I can conceive of nothing in which the people need protection as much as they would from dental quackery. But there are reasons lying at the foundation of the science and history of dentistry, sufficient in my judgment to justify the enactment of the law.

First—Dentistry is a science as well as an art, hoar with antiquity. However useful the village blacksmith, the barber, or the watchmaker may have been in past ages in extracting teeth that day has passed. Possibly, some of the muscular "tooth-extractors" of old, had as much knowledge of the science of dentistry as some of the modern surgeon-dentists, is why you have felt the need of stringent dental laws to regulate its practice. As used and practiced by this Association, I assume that the term dentistry is comprehensive, not confined to the extraction of teeth and the manufacture and fitting in of artificial teeth, but embraces dental physiology, dental pathology, dental therapeutics, mechanical dentistry, metallurgy, etc., as taught and practiced in the highest reputable dental colleges in America.

Second—Dentistry is not the product of modern thought. As an art it appears to have been practiced among that strange and peculiar people, the Egyptians, as a distinct branch of dentistry, at a time "beyond the period of which the memory of man runneth not to the contrary;" and among their ancient tombs the traveler, Belzoni, claimed to have found artificial teeth of wood or ivory fastened upon gold plates. While, therefore, it is not a modern art or science, yet it is modern thought and skill that are carrying it as a science as well as an art, to its highest state of perfection and making it so useful as well as ornamental for dyspeptics of the nineteenth century. A science so useful and beneficial to mankind should not be impeded by charlatans, and the profession brought into disrepute by denticulating humbuggery, and hence the necessity for laws—police protection from the abuse and imposition upon the unsuspecting people. As early as 1843, men devoted to the development of surgery and dentistry, saw the necessity of legislation upon the subject, and appealed to the English Parliament, and obtained what is known as the Medical Act (21 and 22 years' reign Queen Victoria, chapter 90, General Laws) which enabled the profession to unload. This appears to have been the first legislation on the subject, and thereafter it appears that a special examination in dentistry was required of students trained for the profession in the Royal College of Surgeons in England. With the growth and development of this country in the various trades, professions, arts and sciences, and the gullibility of the average American being specially adapted for the growth of quackery of every kind, it is no wonder that laws should have been passed in the different States to regulate the practice of dentistry. Reason and necessity both demanded it.

In the next place, has the Legislature the constitutional right to enact a law to regulate the practice of dentistry? I submit that it has. Such right exists from the very nature of our republican form of government, as a police regulation. The Constitution, Article I., Section 12, among other things, provides that no person shall "be prevented in acquiring, holding, and transmitting property, or hindered in acquiring education, or be liable to any other punishment for any offence, or be subjected in law to any other restraints or disqualification in regard to any personal rights than such as are laid upon others under like circumstances." Judge Cooley, one of the highest authorities on constitutional law, in his excellent work on Constitutional Limitations, on page 713, says: "The police of a State, in a comprehensive sense, embraces its whole system of internal regulation, by which the State seeks not only to preserve the public order, and to prevent offences against the State, but also to establish for the intercourse of citizen with citizen those rules of good manners and good neighborhood which are calculated to prevent a conflict of rights, and to insure to each the uninterrupted enjoyment of his own, so far as is reasonably consistent with a like enjoyment of rights by others." This power cannot be taken from the States or interfered with by the national government, and a proper exercise of it cannot in any way interfere with the general government, and the only constitutional limitations upon the police power of the State, is that no one shall be "liable to any other punishment for any offence, or be subjected in law to any other restraint or disqualification in regard to any personal rights than such as are laid upon others in like circumstances." So long, therefore, as the Legislature in the exercise of the police power, makes the law to fall equally upon all "under like circumstances," then the law is constitutional, unless the fine be "excessive" or the punishment "cruel and unusual," which is a constitutional inhibition.

It must be admitted that the human teeth are living organisms, exceedingly sensitive at times, especially when man is affected with what Burns has so well characterized as "thou hell o' a disease"—perhaps more susceptible to disease and decay than other parts of the body, arising from abuse, constitutional disorder, want of nutrition or vitality of tissue. But who is to diagnose the case so as to get at the root of the matter? The tramp "tooth-extractor," armed with a bottle of patent pain-killer and dirty forceps, or the man skilled in the knowledge of dentistry in its comprehensive sense? As many people are never so well satisfied as when being taken in and humbugged, and are constantly hunting for the cheapest man, the true dentist is often, perhaps, neglected, while the quack gets a job. I insist that the diseases of the teeth and gums, and the proper treatment thereof, is as important to the individual diseased as other branches of medicine and surgery in their respective departments, and is as much open to abuse by the charlatan, and, therefore, is as much entitled to police protection. A man who undertakes and palms himself off as one skilled in dentistry when he knows he is not, and on such representations obtains another man's money for an honest job, when it's only a botch, practices a lie, is a fraud, cheat, and worse than a thief, and should be dealt with accordingly not simply by fine, but by imprisonment at hard labor. Let a negro steal a chicken worth fifteen cents for his supper, he is arrested as a common thief. Let your sleek, oily-tongued "tooth-extractor" come along with his medicine to stop a hundred and one pains, perhaps, that exist only on paper and in the imagination, not worth a fig, but would perhaps work a positive injury to the man who takes it—with his misrepresentations of his work and medicines, he fleeces the man out of a dollar—you put him down as a very smart fellow, when the only difference between him and the negro is, the negro took the chicken for his supper, and the quack took not only the dollar, but the man, too—for a fool—exit, negro to jail—tramp, upon the streets to fleece another fool upon the theory that a fool and his money soon parts. Protection; yes, the country needs it from all professional tramps and swindlers. While your statutes regulating the practice of dentistry have not been before our courts for judical construction, yet the statutes of other States similar in their purpose have been the subject of judical examination. For example, in the State of Arkansas an act was passed providing that dentists practicing within the State, shall, within three months after the passage of the act, obtain a certificate from the Board of Examiners. In the case of Gosnell *vs.* State, this act was sustained as being a proper exercise of the police power of the State and constitutional. 12 Southwestern Reporter, 392.

In Revised Statutes I, by Section 3 of their Act, it is provided that within three months from its passage every person then engaged in practicing dentistry shall be registered with the board of registration in dentistry thereby established, and shall receive from the board a certificate to that effect; and Section 4: "All persons not graduates of regular dental colleges who may thereafter desire to enter the practice of dentistry may appear and be examined * * * and all persons having diplomas from reputable dental colleges may present the same to the board and shall receive certificates without 'examination.' It was held that one practicing dentistry when the Act was passed could not register after the lapse of three months, but that holding a diploma he might apply for a certificate under Section 4, when the board might, if it choose, also allow him to register. Butler vs. Board Reg. (R. I.), 17, A. 131.

In Minnesota, in the case State vs. Vandersluss, 43

North Western Rep. 789, it was held that the laws of Minnesota, 1889, ch. 19, which by Section 5 provide that no one shall have absolute right to be examined for a certificate to practice dentistry who is not possessed of a diploma from some dental college in good standing, the examining board to have the power to determine the standing of the college. Granting the diplomas is not unconstitutional as discriminating against a class who may not have been able to attend a dental college. "The provisions of the Act that it shall not apply to acts of bona fide students of dentistry, done in pursuance of clinical advantages under the direct supervision of a preceptor or a licensed dentist in the State during the period of their enrollment in a dental college and attendance upon a regular uninterrupted course in such college, it is not unconstitutional as discriminating between the students of dentistry as against schools of dentistry of other States. *Ib.*

In the case of the State Board of Dent. vs. People, 13 North Eastern Rep., 201 Hurd, Revised Statutes III, 1885, ch. 91, page 816, was construed: "It is in the discretion of the State Board of Dental Examiners in issuing licenses for the practice of dentistry to graduates of dental colleges to determine what colleges are reputable colleges within the meaning of the Act. But if this discretion is abused from personal or selfish motives to bar the graduates of a particular college from practice, mandamus will lie to compel the issuance of a license. This discretionary power to determine upon the fitness of issuing of license for the practice of dentistry to graduates of reputable dental colleges vested in the State Board of Dental Examiners, cannot be delegated by the State Board to the National Association of Dental Examiners."

In Brown vs. People (Cal.), 17, page 104, it is held that the legislature has power to prescribe what qualifications persons who practice dentistry shall possess, to establish a board of examiners to examine persons who desire to practice that profession, and to prohibit persons who have not received a certificate from such board from practicing."

In case, State vs. Creditor (Kan.), 24 vol., page 346, it was held that the Act regulating the practice of dentistry does not violate Const. U. S., Art. 4, Sec. 2, declaring that "citizens of each State shall be entitled to all privileges and immunities of citizens in the several States," nor Const. U. S., Amend. 14, providing that no State shall make or enforce laws which shall abridge the privilege of immunities of United States citizens.

From the foregoing digest of a few decisions from the States named, it appears that the legislatures of these States in their wisdom, probably in their experience, saw the necessity of protecting their people from the impositions of quacks. The validity of these Acts were tested by such as were unwilling to take their chances before a competent board of examiners and fain would have climbed over the wall, but uniformly have these Acts been sustained by the highest courts of those States on high constitutional grounds involving the construction of the police powers of the State.

In the brief time that I have had to spare from my professional duties to compare our statutes on this subject with those reported from other States, it seems to me that your dental laws are ample, with one exception perhaps. By Section 943 and 2594 the penalty is not less than fifty nor more than three hundred dollars for practicing dentistry in violation of that chapter. If the punishment were a fine or imprisonment for each and every case treated, then I think you would be freed from imposters in your profession. What cares the tramp for a $50 or $300 fine when he has not a dollar to pay with. He can't be imprisoned under the Act. I do not know how far your profession has suffered from charlatans, but I submit that the law is a wise one, and if it applies to all alike in the profession, then no one need claim that it is harsh and infringes upon his liberty to follow what trade suits him best.

I submit that you owe to yourselves as gentlemen of character and reputation to label over the gate of entrance, "None but true men allowed here." You owe it to a profession whose antiquity is coeval with the Pharaohs—to suffering humanity, to have none but men of high character engaged in the business. A man has a right to follow such occupation as he pleases in this country, only in a qualified sense. No man has the right to follow the occupation or profession of a thief, a beggar or a gambler. No man has a right to set himself up for a surgeon unless he is thoroughly acquainted with the anatomy of the human body, and has mechanical skill and judgment. The most virulent poisons are among the best antidotes for certain forms of diseases, but would that justify me in administering strychnine to a sick man? No man has a right to set himself up for a lawyer unless he is familiar with at least a few of the fundamental principles of the law. No man has a right to a living in this world without honest, legitimate work for it. Cheating, gambling and stealing are discountenanced by all laws, human and divine, and the man who gets a living in this world without work, either filches for it, cheats for it or gambles for it out of some one else's labor, unless it is given to him, and that is contrary to God's moral law. Show me a man who never earned an honest penny in some honest way, and I will show you a fool or a knave. Any man, therefore, who follows a trade or profession and demands pay for his work, should be able to do the work for which he demanded pay, properly and honestly. This applies to the dentist as well as to the lawyer, doctor or mechanic. In trades and mechanics the people are not so easily imposed upon, but in the learned professions of law and medicine, the people, through their representatives, have demanded laws to protect them from charlatans and quacks, and the demand applies to dentists as well as to the lawyer and doctor.

One great trouble, perhaps the greatest drawback to the learned profession is, that they are followed for the money that can be gotten out of them, rather than for the ends of justice or the alleviation of human misery. Man's selfishness has made law a necessity. It is the motive power of all man's infamy. It prompts him to nigh cuts in getting money in professions as well as in trades. Necessity demands that the law should closely watch the gates and build the walls so high that thieves and robbers can climb over them.

Then there is another branch of dental law to which I will briefly call your attention. You are a dentist in the true sense of the word, regularly licensed and engaged in the practice. What is required of you in the practice of your profession? I answer, you are required to have a reasonable degree of skill and care in your professional operations—you are not to be answerable for injuries arising from the want of the highest attainments in your profession. It has been held that a dentist in using chloroform or other anæsthetic agent, is only bound to look to the natural and probable effects, and not answerable for results arising from the peculiar condition or temperament of the patient of which he had no knowledge. "Every person who enters into a learned profession undertakes to bring to the exercise of it a reasonable degree of care and skill. He does not undertake, if he is an attorney, that at all events you shall gain your cause; nor does the surgeon impliedly undertake that he will perform a cure, nor does he undertake to use the highest possible degree of skill, but he undertakes to bring a fair and competent degree of skill," is the language used by an eminent jurist and quoted with approval by text-writers. (1 Ad. on Torts, 569).

This rule applies as well to the dentist as to the surgeon. Therefore, if a dentist undertake an operation or the treatment of a disease in the line of his profession, he "undertakes to bring a fair and competent degree of skill" to his aid, and if he should perform the operation in a negligent manner and without a competent degree of skill, and injury to the health of the patient was the result of his negligence, then he would be liable in damages. So also in the administration of chloroform or other anæsthetics, a dentist is inexcusable if he does not look to the natural and probable effects that it would have on the patient under ordinary conditions. This, in brief, is as I understand the law, applicable to a dentist in the practice, and is founded upon reason and common sense—principles of the common law independent of any statutory regulations.

In conclusion, Mr. President, let me suggest: No man familiar with the leading principles of the profession, and having practical knowledge and ordinary skill in the uses of the instruments of his profession, need hesitate to appear before a board of gentlemen for examination, provided he is an upright and honest man. He ought not to fall out with the law which requires him to show beforehand that requisite knowledge and skill. This is the law, and it is the duty of every honest man to obey that law so long as it falls upon others alike in the same circumstances. It is the man who constantly violates the laws of his country who despises those laws, while the law-abiding citizen looks to those laws with pride and veneration for his protection, and he naturally looks with suspicion, and justly, too, upon him who tries to outrun or evade the law at every turn of the road.

We are grateful for all of these, and for the presence of the fair ones here, as ever in their beauty and loveliness; believe me, gentlemen, or disprove it if you can, the warp and norp of the character of man is ever laid by woman's hand.

Dr. Beach, of Clarksville, Tenn. (Treasurer): I believe, sir, it is one of the fundamental features of our organization, that all members, before they shall be allowed any privilege on the floor, should pay their dues. The dues will be $3 instead of $5, as heretofore. After the collection the president appointed Drs. B. A. Muckinfuss, South Carolina; Jas. J. Johnston, Virginia; J. S. Thompson,

Georgia; H. M. Morgan, Tennessee, and H. C. Herring, North Carolina; on executive committee, to take the places of absentees.

Dr. Beadles, of Virginia, moved that the reading of the minutes be dispensed with. The motion carried.

The president: We will now hear the report of the executive committee.

The executive committee asked for a little more time, which was granted them.

The president: The committee of arrangements will now report.

Dr. Turner, chairman of committee of arrangements: It has been suggested that the printed order of business, as suggested by the president, should be changed somewhat, in order to have the clinics all in one day. I believe the experience of the dental association generally, has been that clinics are frequently interrupted by the call of the house, and in order to avoid such confusion and inconvenience to clinic operators, it has been decided to propose, that we devote to-morrow (Aug. 12) to clinics. Should there be any clinics necessary to be finished, we might provide some hour on the following day for that.

I have nothing else to suggest, except that on Friday (Aug. 14) we have a morning and evening session. It is proposed to have a night session; that is a matter for discussion. I don't know whether we will be able to maintain our whole force here, but we propose that this day shall be devoted entirely to reading the report of committees, and to morrow be devoted to clinics. We will adhere to this arrangement until tomorrow.

---

Dr. V. E. Turner, North Carolina: I move that we have an afternoon session to day, at 3:30, and a night session at 8:30. The afternoon session to adjourn at 5:30. I suggest that to-morrow afternoon the Association have a holiday, because the clinics will not be over by the afternoon. Adopted.

Dr. B. A. Muckinfuss, Chairman of the Executive Committee, reported two names for membership, Dr. E. J. Quattlebam, S. C., and Dr. D. D. Lester, Va.

Dr. Johnston moved that the rules be suspended and the secretary instructed to cast ballot of the Association in favor of these gentlemen.

Dr. Beadles moved to adjourn to meet at 3:30. Carried.

*To be continued.*

